# Supplementary material for: Implantable Cardioverter Defibrillator Multisensor Monitoring during Home Confinement Caused by the COVID-19 Pandemic
Source: Biology (Basel). 2022 Jan 12;11(1):120. doi: 10.3390/biology11010120 (PMC8772791; doi:10.3390/biology11010120)
Supplement: Supplementary file 1 [file biology-11-00120-s001.zip › biology-1529522-supplementary.pdf]

Full list of participant centers and investigators

- Institute of Cardiology, University of Bologna, S.Orsola-Malpighi University Hospital, Bologna, Italy: Ziacchi M., Diemberger I, Biffi M., Cristian Martignani, Andrea Angeletti, Giulia Massaro, Giovanni Statuto.
- Policlinico Casilino, Rome, Italy: Calò L., De Ruvo E., Martino A, Panucci M.
- Unità Operativa di Elettrofisiologia, Studio e Terapia delle Aritmie", Monaldi Hospital, Naples, Italy: D'Onofrio A., Bianchi V., Tavoletta V.
- OO.RR. San Giovanni di Dio Ruggi d'Aragona, Salerno, Italy: Manzo M., Esposito C., Franculli F.
- Clinica di Cardiologia e Aritmologia, Università Politecnica delle Marche, "Ospedali Riuniti", Ancona, Italy: Dello Russo A., Guerra F., Casella M., Molini S.
- "Giovan Battista Grassi" Hospital, Rome, Italy: Santini L., Ammirati F., Mahfouz K., Colaiaco C.
- "F. Spaziani" Hospital, Frosinone, Italy: Giubilato G., Carbonardi L.
- Azienda Ospedaliera Universitaria Ospedali Riuniti di Trieste – Cattinara, Trieste, Italy: Carriere C., Sinagra G., Zorzin Fantasia A., Zecchin M.
- University of Bari, Policlinico di Bari, Bari, Italy: Santobuono V.E., Favale S., Guaricci I.
- S. Giovanni Battista Hospital, Foligno, Italy: Savarese G.
- Fondazione Poliambulanza, Brescia, Italy: La Greca C., Pecora D.
- Ospedale Civile Apuane, Massa, Italy: Arena G., Bartoli C., Borrello V. M., Ratti M.
- SS. Annunziata Hospital, Cosenza, Italy: Talarico A.
- Vito Fazzi Hospital, Lecce, Italy: Pisanò E., Milanese G., Lauretti M., De Leonardis M.
- Division of Cardiology, Maria Vittoria Hospital, Turin, Italy; Giammaria M., Amellone C., Lucciola M. T., Ebrille E.

- “Bianchi-Melacrino-Morelli” Hospital, Reggio Calabria, Italy: Pangallo A., Benedetto F.
- Sacro Cuore Don Calabria Hospital, Negrar (VR), Italy: Molon G., Costa A.
- “Carlo Poma” Hospital, Mantova, Italy: Pepi P., Nicolis, D.
- S. Anna e S. Sebastiano Hospital, Caserta; Italy: Viscusi M., Brignoli M., Mattera A.
- S. Pietro Fatebenefratelli Hospital, Rome, Italy: Porcelli D., Romani B.
